# Sensory augmentation: integration of an auditory compass signal into human perception of space

**DOI:** 10.1038/srep42197

**Published:** 2017-02-14

**Authors:** Frank Schumann, J. Kevin O’Regan

**Affiliations:** 1 Laboratoire Psychologie de la Perception – CNRS UMR 8242, Université Paris Descartes, Paris, France

## Abstract

Bio-mimetic approaches to restoring sensory function show great promise in that they rapidly produce perceptual experience, but have the disadvantage of being invasive. In contrast, sensory substitution approaches are non-invasive, but may lead to cognitive rather than perceptual experience. Here we introduce a new non-invasive approach that leads to fast and truly perceptual experience like bio-mimetic techniques. Instead of building on existing circuits at the neural level as done in bio-mimetics, we piggy-back on sensorimotor contingencies at the stimulus level. We convey head orientation to geomagnetic North, a reliable spatial relation not normally sensed by humans, by mimicking sensorimotor contingencies of distal sounds via head-related transfer functions. We demonstrate rapid and long-lasting integration into the perception of self-rotation. Short training with amplified or reduced rotation gain in the magnetic signal can expand or compress the perceived extent of vestibular self-rotation, even with the magnetic signal absent in the test. We argue that it is the reliability of the magnetic signal that allows vestibular spatial recalibration, and the coding scheme mimicking sensorimotor contingencies of distal sounds that permits fast integration. Hence we propose that contingency-mimetic feedback has great potential for creating sensory augmentation devices that achieve fast and genuinely perceptual experiences.

Starting from the seminal work of Bach-y-Rita^1^, sensory substitution and augmentation research has aimed to restore sensory functionality from non-invasive afferent signals of artificial sensors. However, as noted in an extensive recent review by Deroy & Auvray^2^, there has been little concrete evidence that truly perceptual experiences have ever been obtained via this approach. Evidence robustly shows abilities to locate and identify objects and shapes using sensory substitution devices^3^,^4^,^5^,^6^, yet these abilities are typically constrained to a small number of (known) stimuli and do not match the speed or the accuracy of natural perceptual object identification^2^. Thus, it is debatable whether they really involve perceptual experiences similar to the source modality, or are based on higher-level cognitive decision strategies for stimulus discrimination. Deroy & Auvray conclude that the skills achieved with sensory substitution devices should not be interpreted as being ‘perceptual’ but rather should be described as “acquired cognitive extensions to existing perceptual skills”^2^. This seems similarly to be the case for approaches to sensory augmentation which reduce the complexity of the artificial afferents by using simpler artificial sensors^6^,^7^. For instance in the case of magnetic North, behavioural integration has been obtained in rats using neuroprosthetics^8^. However, in humans using sensory augmentation König *et al*.^9^ could not demonstrate low-level sensory effects using a vibro-tactile approach, and subjective reports indicate high cognitive involvement as described for sensory substitution^2^,^9^,^10^.

Why has it, beyond stimulus discrimination abilities, not been possible by using sensory substitution and augmentation to create truly modal perceptual experience that (begins to) resemble that of a natural modality? A key to creating perceptual sensations from an artificial afferent signal seems to be that the signal should find an ‘entry point’ to interface with sensory processes that are already in place^11^. Yet many classical approaches to sensory substitution have contacted the sensory apparatus with more or less arbitrary sensory coding schemes that do not necessarily have a direct correspondence to the low-level processes of the source modality. This is the case even when the codes are based on genetically, statistically or culturally established analogies or associations^12^,^13^. As a consequence, an entry point into existing sensory processing might have to be established via perceptual learning and cross-modal neural plasticity^2^,^12^,^14^,^15^,^16^, for instance by a mechanism proposed in a recent cross-modal extension to reversed hierarchy theory^11^,^17^. However, perceptual learning typically requires hundreds and thousands of trials of training for changes in perception to occur^11^,^18^, if it occurs at all. Indeed, in the case of visual sensory substitution, Arno *et al*.^5^ and Collingon *et al*.^19^ showed no neuro-anatomical evidence for cross-modal plasticity over the time period of their experiment. And in early blind participants, where plasticity has been observed, it is likely explained as a rewiring due to (a life-time of) sensory loss rather than as resulting from the usage of a substitution device^5^,^20^,^21^. Hence from a learning perspective, it may thus not be surprising that Deroy & Auvray’s^2^ review does not find reports of genuinely perceptual experience when using a sensory substitution device, where training generally involves only a few hours of learning. Instead, users report engaging in explicit higher-level cognitive decision strategies during learning and when using substitution devices^2^, even in cases of extended training for months or years^2^,^22^.

On the other hand, creating perceptual experience from artificial sensors has recently been possible in the field of brain-machine-interfaces (BMI). BMI systems interface artificial sensors with the sensory apparatus using intra-cortical microstimulation via implanted electrodes, for instance to convey somatosensory feedback about the movement of a neuro-prosthesis^23^,^24^, or more recently also via mechano-neuro-transduction (MNT)^25^. Like their non-invasive counterparts, invasive interfaces also require the design of an adequate coding scheme to ‘enter’ the already existing sensory processing stream. In the BMI context, this can be achieved by learning artificial neural codes, but also here tremendous amounts of training are generally required^26^. Fast learning of perceptual sensations, however, for instance from artificial signals about the pressure of touch^23^,^24^,^27^ or texture at an artificial fingertip^25^, has been possible in BMI approaches that use a coding scheme that seeks to approximate the primary neural sensory signals arising in normal behavior, termed bio-mimetics.

Here we test if the mimetic principle can be transferred to non-invasive sensory augmentation, and equally lead to fast acquisition of perceptual experience. In analogy to biomimetic BMI, we transmit the output of an electronic sensor to the sensory apparatus using a coding scheme that mimics the natural characteristics of the interfacing modality. But we apply the principle of biomimicry at the sensor rather than the neural level, which we in analogy term “contingency-mimetic” sensory augmentation. As a demonstration of the principle, we developed a device that provides information about the orientation of the head relative to geomagnetic North by mimicking the sensorimotor contingencies^28^ of distal sounds using auditory head-related transfer functions (HRTF)^29^. This approach directly mimics natural sensory features of the interface modality, here the sensory contingencies of distal sounds coming from a particular direction. Using a self-rotation experiment, we show that a geomagnetic signal conveyed via auditory contingency-mimetic coding can be integrated into the perception of space.

## Results

**Figure 1:**
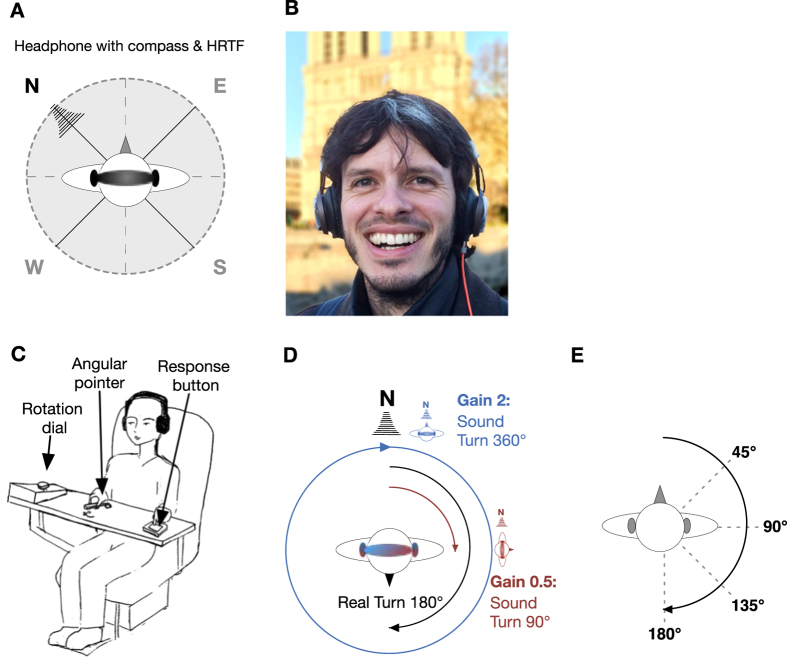
Setup & Experimental Protocol. **(A,B)** The hearSpace app. Headphones with incorporated orientation sensors (compass, gyro, accelerometer) measure the orientation of the head relative to geomagnetic North. An iPhone connected to the headphone translates this information into a waterfall sound reliably coming from North using the head set’s sound engine based on average adult head related transfer functions that approximate the contingencies with which external sounds are filtered differently by pinnae, head, shoulder and torso geometry depending on their direction. The novel magnetic directional information is thus naturally processed as a distal spatial signal. **(C)** Rotation Chair & Angular Pointer. Blindfolded participants were seated on a motorized rotating chair that could be controlled by the experiment computer or actively by participants themselves via a rotation dial that governed the velocity of the chair. The rotation dial and an angular pointer that was used to indicate the size of a turn during the experiment were mounted on a board situated in front of participants. **(D)** Magnetic-Vestibular Adaptation Training. To test effects of the magnetic augmentation signal on the perception of space, we introduced a conflict between real rotations and the magnetic signal on the headphones. During a short training period, blind-folded participants seated on the rotation chair performed passive and active rotations in the horizontal plane of always 180°. For those participants training with a gain of 0.5 (red line), the compensatory movement of the waterfall sound would indicate a self-rotation of only 90°. For participants training with a gain of 2 (blue line), the sound would indicate a self-rotation of full 360°. **(E)** Self-Rotation Tests. Before and after training, we measured participants’ vestibular-only estimate of the extent of passive self-rotations on the rotation chair. The blindfolded participants were turned passively through different angles and asked to turn an angular pointer back to the direction from which the rotation started. Importantly, the augmentation signal was switched off during the test.

Our novel iPhone based sensory augmentation device measures head orientation to North via orientation sensors (compass, gyro, accelerometer) integrated into a headphone and transforms their output into a spatial sound using a sound engine based on head-related transfer functions (HRTF) (Fig. 1A,B). A recording of a waterfall serves as the sound source which provides the ecological semantics of a natural sound coming from a distance. Further, the sound has a pink-noise like frequency spectrum which is pleasant to hear^30^. The waterfall sound is reliably situated in the direction of magnetic North, moving in such a way as to compensate the movements of the head. This artificial sensorimotor contingency^28^: (1) allows aligning the head with a global reference, creating a reliably stable artificial external reference for the eyes, ears and the vestibular system, and (2) provides an intuitive sensory code that mimics the acoustic characteristics of distal sounds.

### Experiment 1

#### Training

**Figure 2:**
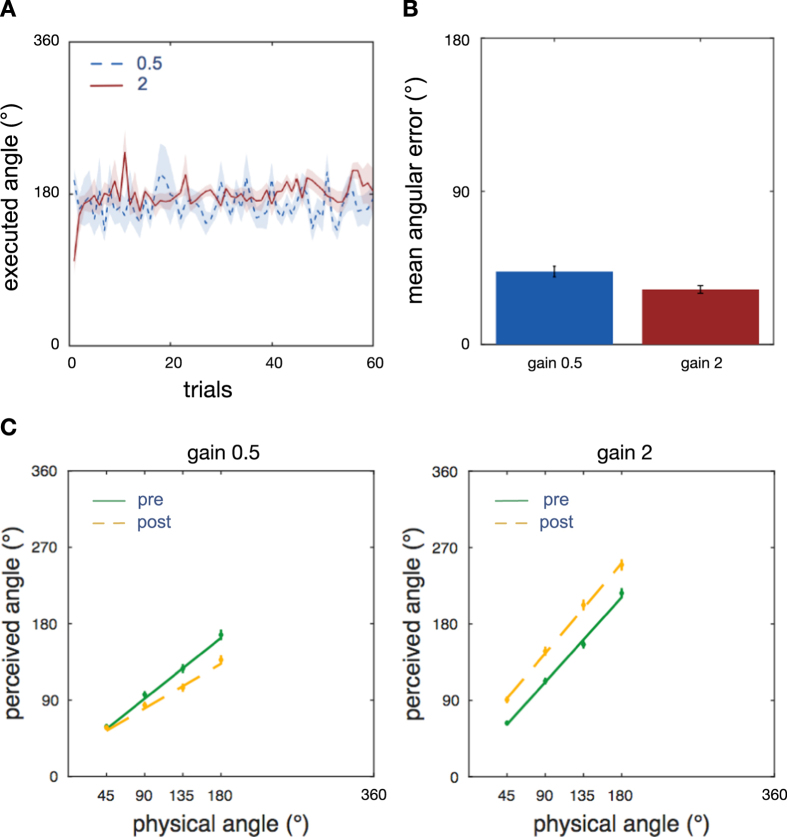
Results Experiment 1. **(A)** Trial-by-Trial Training Accuracy. Average of rotation magnitudes when using the sound to actively rotate through 90° (gain 0.5) and 360° (gain 2) in virtual space, both corresponding to 180° in real-world space during active training blocks. Both turning directions plotted as positive values. The blue line shows training with gain 0.5 (mean over 14 subjects), the red line training with gain 2 (mean over 13 subjects). The shaded regions show the respective standard error of the mean (sem). **(B)** Average Training Accuracy. The average accuracy of active rotations for each gain group shows that participants used the sound to accurately accomplish the training task, confirming that they heard the sound as a spatial signal. Participants were slightly more accurate with the larger gain factor of 2. Error bars depict sem (over participants). **(C)** Perceived Self-Rotation. Training with a magnifying or minifying gain conflict to the virtual relation to North induced an expansion or compression of the perceived extent of self-rotation after the training, even though the augmenting signal was absent during the test.

To test if intuitive sensory access to an artificial magnetic spatial reference can change the perception of space, we trained blindfolded participants seated on a motorized rotation chair in a darkened room in a situation of sensory conflict with the magnetic North information. We modified the magnetic contingency by applying a gain factor of either 0.5 or 2 between the magnetic-auditory signal and real rotations (Fig. 1D). Participants always performed real body rotations with a magnitude of 180° on the rotating chair, while the auditory signal provided virtual cues signalling smaller (90°) or larger (360°) rotations. Training involved consecutive blocks of passive and active rotations. Passive rotations were controlled by the computer and followed a triangular velocity profile with an acceleration of 11°/s^2^. Active rotations were governed by participants themselves using a rotating dial in front of them that controlled the rotational velocity of the chair. Participants rotated so that the sound, in the modified virtual space, moved leftward or rightward by either 90° or 360°. For instance, if the rotation began with the sound in front, participants training with the compressing gain factor were asked to rotate themselves until they heard the sound coming from the left or the right, corresponding to a 90° turn in the virtual magnetic space and a turn of 180° in real world space. By contrast, participants training with the expanding gain factor were asked to rotate until they heard the sound again as coming from the front, corresponding to a 360° turn in virtual sound space but equally to a 180° turn in real world space. In this gain 2 condition, the starting location by necessity was kept identical within, but differed across the active training blocks. Each new trial started at the end position of the previous trial. Passive and active training blocks switched after 7 minutes, and in total, participants trained for either 200 trials or 45 minutes, whichever came first. This led on average to 144 training trials per participant (std = 28, range 110–200). Despite the conflicting gain, participants executed the training rotations accurately in real-world space, with an error slightly larger when training with gain 0.5 than with gain 2 (gain 0.5: mean = 43°, std = 11.2°, across subjects; gain 2: mean = 32°, std = 8.5°; two-sample t-test, t(25) = 2.78, p = 0.01, Fig. 2A,B). Since participants were asked to rotate using the magnetic sound and had no knowledge of the required real-world rotation, this confirms that they perceived the magnetic-auditory signal as an external spatial sound which they used to accomplish the task. It is also consistent with participants’ reports after the experiment that they relied on the magnetic sound to execute the turns with the desired magnitude, and with the larger error when training with gain 0.5 (since an error in the real world angle = error in the sound angle/gain). Further, the majority of participants reported the sound as coming from a stable direction, and themselves as turning in front of it. Only two of the twenty-seven participants (both in the gain 2 group, and none in the gain 0.5 group) reported that the sound moved around them. This indicates that the auditory North signal was not only perceived as a spatial sound, but as being a stable external reference.

#### Perception of Self-Rotation

We examined perceptual integration of the magnetic spatial reference by measuring the perceived magnitude of vestibular-only self-rotations before and after training in the absence of the auditory augmentation signal. While hearing white noise on the headphones, participants were rotated passively on the chair through different angles (45°, 90°, 135° and 180°) and were asked to indicate the perceived size of each rotation using an angular pointer placed in front of them (see Fig. 1C). Linear mixed effect models (equation 1) revealed that participants’ angular estimations before training are a linear function of the rotation angle (gain 0.5: intercept 20.35°, 95% CI [10.12 30.57], t(1912) = 3.9, p < 0.001, slope 0.79, 95% CI [0.58 1.00], t(1912) = 7.3, p < 0.001; gain 2: intercept 11.8°, 95% CI [1.41 22.32], t(2201) = 2.22, p = 0.02, slope 1.10, 95% CI [0.93 1.28], t(2201) = 12.27, p < 0.0001, Table 1; N.B. slope difference between groups = 0.31 (t(2074) = 2.2, 95% CI = [0.04 0.58], p = 0.02). This replicates prior results on passive vestibular self-rotation in the dark^31^. When comparing the post-training judgements to the baseline of each group, we find that after exposure to the conflicting augmenting orientation signal during the training, self-rotation estimates decrease or increase, respectively (gain 0.5: slope difference = −0.21, 95% CI [−0.32–0.1], t(1912) = −3.74, p < 0.001; gain 2: intercept difference 27.5°, t(2201) = 4.23, 95% CI [14.78 40.28], p < 0.001). Importantly, this is despite the fact that the auditory signal was absent in the tests, suggesting that training with the auditory signal recalibrated participant’s vestibular spatial perception of self-rotation (Fig. 2C). The groups differed in the types of effect. The gain 0.5 group showed a reduction in the slope of the model after training, i.e. larger reduction of perceived rotations with larger rotations. In the gain 2 group, we did not find a corresponding difference in slope (slope difference = 0.07, t(2201) = 1.17, 95% CI [−0.04 0.18], p = 0.16), but a larger intercept after training, i.e. a larger expansion of perceived rotations independent of the size of the rotation, which was not found in the gain 0.5 group (intercept change = 7.48, t(1912) = 7.4, 95% CI [−2.15 17.12] p = 0.23). Model comparison with a theoretical likelihood ratio test revealed main effects of time (post training to baseline) on response angles in both groups when removing the combined intercept and interaction as fixed effects from the respective model (gain 0.5: LRS(3) = 23, p < 0.0001; gain 2: LRS(3) = 34.56, p < 0.0001).

### Experiment 2

In experiment 2, we controlled for the possibility that in experiment 1 participants did not use vestibular information to determine their turning angle, but rather used a strategy based on the duration of the turns. To do this, we increased the number of stimulus angles and varied the velocity profiles of each turn^31^,^32^, presented in random order. Additionally, we reduced the training duration by almost half. Lastly, we measured both gain conditions within each subject, randomised across participants, by running the full experiment (pre-training, training and post-training) twice, on different days, and examined if the recalibration on day 1 was long-lasting and affected pre-training on day 2.

#### Training

**Figure 3:**
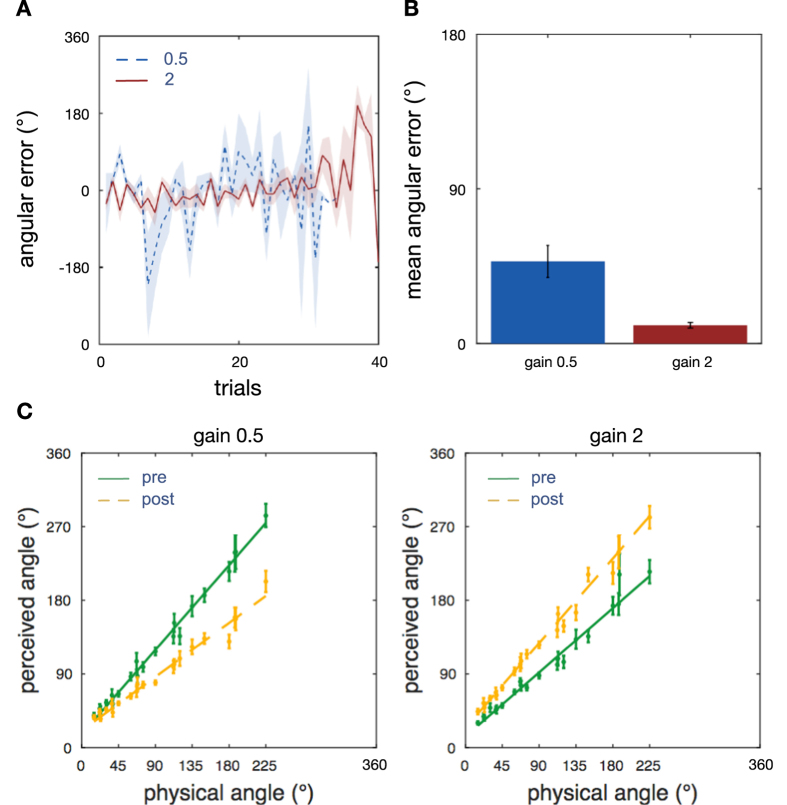
Results Experiment 2. Experiment 2 is identical to Experiment 1, but used an increased number of stimulus angles, varied the velocity profiles of each turn, and measured both gain conditions within each subject, randomised across participants and on separate testing days. **(A)** Trial-by-Trial Training Accuracy. Average rotation errors when using the sound to actively rotate through a larger set of real-world angles (45° to 360° in steps of 45°) during active training blocks. The shaded regions show sem (over 15 subjects). **(B)** Average Training Accuracy. Same as in Fig. 2B. Results confirm that participants could use the sound to accurately accomplish the training task even with a larger set of target angles, and thus heard the sound as a spatial signal. **(C)** Perceived Self-Rotation. Same as in Fig. 2C, except that the set of rotation stimuli was larger and varied the velocity profiles (maximum velocities of 30°/s, 45°/s, 60°/s, 75°/s, accelerations of 23°/s^2^, 46°/s^2^, 69°/s^2^, 92°/s^2^, 138°/s^2^, 161°/s^2^, displacement range of 15–225°). Results confirm that training with the virtual relation to North induced expansion or compression of the perceived extent of self-rotation after the training, even though the augmenting signal was absent during the test.

On each of the two testing days, we trained participants with an increased number of eight rotations magnitudes from 45° to 360° in steps of 45°. Passive training rotations were constructed with trapezoidal velocity profiles of peak velocities 30°/s, 45°/s, 60°/s and 75°/s and accelerations of 23°/s^2^, 46°/s^2^, 69°/s^2^ and 113°/s^2^. In the active training rotations, participants were instructed to rotate themselves towards one of the compass directions. They were instructed that they would hear the sound being in front of them when facing ‘North’, behind at ‘South’, to the right at ‘East’, to the left at ‘West’, and accordingly for the oblique directions ‘North-East’, ‘North-West’, etc. We reduced the total training time to 25 minutes and switched passive and active training blocks after 5 minutes. This led on average to 107 training trials per participant (std = 20). As in experiment 1, participants executed the training rotations accurately with an error larger when training with gain 0.5 than with gain 2 (gain 0.5: mean = 47.8°, sem = 9.3°, across participants; gain 2: mean = 10.65°, sem = 1.6°; two-sample t-test, t(28) = 3.9, p < 0.001, Fig. 3A,B). In the gain 2 condition active training rotations were more accurate than in experiment 1, while in the gain 0.5 condition performance had a similar accuracy (Figs 2B and 3B). To acquaint participants with the active turns, they were instructed to move to cardinal directions only in the first 24 trials of the training. This explains the smaller sem compared to the subsequent trials that included the oblique directions of the compass (Fig. 3A). It is surprising that increasing the number of rotation angles resulted in more accurate active training performance than in experiment 1. Since participants again had no knowledge of the required real-world rotation, this further confirms that they perceived the magnetic-auditory signal as a stable external spatial sound which they used to accomplish the task. And indeed, again only a small number of participants reported the sound as sometimes moving around themselves (two in the gain 0.5 and one in the gain 2 condition).

#### Perception of Self-Rotation

Before and after the training of each testing day, we again tested the changes in participants perceived self-rotation, but also using a larger set of rotation magnitudes and velocity profiles than in experiment 1. Stimuli had trapezoidal velocity profiles with maximum velocities of 30°/s, 45°/s, 60°/s, 75°/s, accelerations of 23°/s^2^, 46°/s^2^, 69°/s^2^, 92°/s^2^, 138°/s^2^, 161°/s^2^ and displacement range of 15–225°. Otherwise the experiment was conducted identically to experiment 1. As in experiment 1, participants’ angular estimations before training are a linear function of the rotation angle even under varying velocity profiles (gain 0.5: intercept 15.97°, 95% CI [4.03 27.92], t(1412) = 2.62, p < 0.009, slope 1.15, 95% CI [0.99 1.29], t(1412) = 14.98, p < 0.0001; gain 2: intercept 13.51°, 95% CI [6.89 20.13], t(1412) = 4.000, p = 0.009, slope 0.87, 95% CI [0.72 1.01], t(1412) = 11.69, p < 0.0001, Table 2). Results further confirmed that self-rotation estimates decrease or increase after exposure to the conflicting augmenting orientation signal also with a stimuli set that counteracts counting strategies, and again despite the fact that the auditory signal is absent (Fig. 3C). Unlike in experiment 1, in experiment 2 both gain groups showed a change in the slope of the linear-mixed effect model (gain 0.5: slope difference = −0.41, 95% CI [−0.53–0.29], t(1412) = −6.8, p < 0.001; gain 2: slope difference = 0.27, t(1412) = 4.99, 95% CI [0.16 0.38], p < 0.001). As in experiment 1, the gain 2 condition also shows a slight increase in the intercept that was not observed in the gain 0.5 condition (gain 2: intercept difference 10.6°, t(1412) = 2.37, 95% CI [1.86 19.47], p < 0.02; gain 0.5: intercept difference 4.5°, t(1412) = 0.9, 95% CI [−5.34 14.54], p = 0.36). Furthermore, model comparison with a theoretical likelihood ratio test also confirms the main effects of time (post training to baseline) on response angles when removing the combined intercept and interaction as fixed effects from the respective model (gain 0.5: LRS(3) = 42.60, p < 0.0001; gain 2: LRS(3) = 45.26, p < 0.0001).

**Figure 4:**
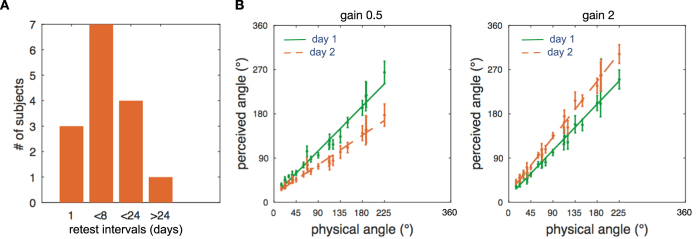
Long-Lasting Recalibration Effects in Experiment 2. **(A)** Number of days that passed between testing day 1 and testing day 2 (median 5 days) in experiment 2. **(B)** The comparison of pre-training rotation estimates between both days shows that vestibular recalibration established by the training on day 1 persisted and affected the pre-training rotation estimates measured on day 2, depending on the training gain on day 1. This demonstrates a long-lasting vestibular recalibration effect from training with our novel magnetic-auditory sensory augmentation device.

Next we analyzed if recalibration was long-lasting, i.e. whether the training on day 1 affected the pre-training baseline judgements on day 2. Most measurements on day 2 occurred within the same week, and some up to 3 weeks after day 1 (median 5 days, Fig. 4A). We fitted separate linear-mixed effect models for each training gain on day 1 comparing the pre-training baseline performance on day 1 with that of day 2 (equation 2). As expected, on day 1, prior to any training, rotational judgements in the baseline measurements are very similar, with a slope close to 1 in both groups (gain 0.5: baseline slope day 1 = 1.01, 95% CI [−0.83–1.19], t(656) = 11.42, p < 0.0001; gain 2: baseline slope day 1 = 1.04, t(754) = 13.03, 95% CI [0.88 1.20], p < 0.0001, Table 3), and also both intercepts show a similar slightly positive bias (gain 0.5: baseline intercept day 1 = 13.59°, t(656) = 1.93, 95% CI [−0.21 27.41], p < 0.05; gain 2: baseline intercept day 1 = 11.53°, t(754) = 2.0, 95% CI [−0.26 22.8], p = 0.04, Table 3). Thus on day 1, prior to any training, subjects on average had a surprisingly veridical angular perception of rotations with a slope close to 1 and only a slight bias. By contrast, we found that on testing day 2, the slope of the pre-training baseline performance was no longer veridical but remained significantly smaller after training with gain 0.5 on day 1, and significantly larger after prior training with gain 2 (Fig. 4B, gain 0.5: slope difference =−0.34, 95% CI [−0.50–0.18], t(656) = −4.1, p < 0.001; gain 2: slope difference =0.22, t(754) = 4.99, 95% CI [0.08 0.36], p = 0.002, Table 3). This long-term recalibration selectively affected only the slope of the pre-training judgements on day 2, but not the intercept (gain 0.5: intercept difference =2.4°, t(656) = 0.37, 95% CI [−10.24 14.96], p < 0.71; gain 2: intercept difference = 6.43°, t(754) = 0.9, 95% CI [−9.68 22.56], p = 0.43, Table 3). This demonstrates a long-lasting recalibration depending on the training gain that is selectively proportional to the magnitude of the angular turning, but not leading to a bias. Further, the long-lasting recalibration affecting the baseline at day 2 had a similar size as the recalibration observed in the post-training measure of day 1 (gain 0.5: post-training slope day 1 = −0.41, baseline at day 2 = −0.34; gain 2: post-training slope day 1 = 0.28, baseline at day 2 = 0.22). In summary, experiment 2 demonstrates rapid and long-term recalibration of vestibular self-rotation judgements following training with our novel magnetic-auditory directional sensory augmentation signal.

## Discussion

Our results demonstrate rapid and long-lasting vestibular recalibration with a sensory augmentation device signaling the direction of geomagnetic North. The observed changes in self-rotation estimates cannot result from a cognitive mode of using the augmentation device, since the signal was switched off in the test. The effects can also not be explained by general training effects such as a training-induced loss in vestibular sensitivity which might have led to a compression of angular judgements^33^,^34^, since we also observe an opposing expansion of perceived self-rotations with the gain 2 manipulation. Further, using a variable stimulus set excludes a possible interpretation of the results in terms of duration counting strategies^31^,^32^. Our results therefore suggest a genuine change in the perception of space, as reflected by persistent vestibular re-calibration^35^, induced by an auditory augmentation signal indicating magnetic North.

We thus present a novel method of sensory augmentation that leads to fast acquisition of perceptual experience from an artificial afferent signal. Like biomimetic brain-machine interfaces^23^,^24^, our novel approach translates the output of a sensor using a coding scheme that mimics the natural characteristics of the interfacing modality, but does so by applying the principle of biomimicry non-invasively at the sensor rather than the neural level, which we term contingency-mimetics. We demonstrate this approach by creating a magnetic-to-auditory augmentation signal that has two aspects: (1) The higher-order magnetic aspect of the contingency that is provided by the electronic compass and establishes the unique novel allocentric spatial stability that we wished to integrate into spatial perception; (2) The lower-order sensory aspect of the contingency that is provided through the use of HRTF: The HRTF describes the spectral characteristics with which external sounds arriving from a specific direction are filtered by pinnae, head, shoulder and torso^29^, and hence creates a sensory interface that mimics the acoustic signals of spatial sound coming from a particular direction. This approach thus piggy-backs the ‘magnetic’ information of a head-based compass on sensory cues of distal sounds. The magnetic spatial information can this way interface with existing spatial processes via natural mechanisms of auditory localization. Contrary to tactile waist-stimulation approaches^9^,^36^,^37^,^38^,^39^, additionally, the magnetic information is presented close to real time and shares the reference frame of the eyes, ears and vestibular equilibration, favoring temporal and spatial integration^39^,^40^,^41^,^42^.

In contrast to this novel biomimetic approach to sensory augmentation, a basic assumption of sensory substitution and augmentation has been that perception is to some degree independent of particular lower-level sensory information^43^,^44^,^45^,^46^, allowing to substitute signals of one modality via another, or to augment perception with signals from novel sensors. This assumed independence is largely grounded in theories according to which perceptual processing is organized in meta-modal structures involving computational principles that are independent of a given modality. Meta-modal theories pose that what is important for perception to occur is that the sensory signals contain relevant meta-modal structural information such as spatial information, or information about shape^18^,^46^,^47^,^48^, but not (all of) the particular lower-level features in which this information is conveyed.

Notwithstanding meta-modal theories, however, a large body of research on sensory substitution has so far been unable to convincingly demonstrate the creation of properly modal perceptual experience from the meta-modal information conveyed in the afferent signals provided by such devices^2^. Even for those devices that base their coding schemes in part on cross-modal correspondences between the modalities^12^,^13^ and lead to clear improvements in forced choice stimulus discrimination^49^, it remains debatable if these improvements demonstrate genuine perceptual experiences^2^. Similarly, a recent approach to providing sensory augmentation like we did with spatial information about magnetic North, except using tactile stimulation instead of auditory, indicated high cognitive involvement in interpreting the signal, and did not produce measurable non-cognitive perceptual changes to the experience of space^9^. Against this background, our results suggest that the meta-modal assumption, at least if taken to the extreme, is likely to be false – and that therefore lower-level sensory contingencies might be required for creating perceptual experience with modal quality.

An exclusively meta-modal perceptual process is indeed questioned by neurophysiological evidence. While perception has been shown to correlate with processing in higher-level neural areas^50^, as suggested by meta-modal theories, reciprocal connections from these higher-level areas also massively shape processing in early sensory areas, indicating that lower-level sensory features may be necessary to establish a modal percept^51^,^52^. And in some cases, activity in early sensory areas directly correlates with what is perceived^53^,^54^, a finding that cannot easily be explained in a solely meta-modal account. Hence, meta-modal information provided by a substitution or augmentation device using an arbitrary sensory coding scheme might relatively quickly become part of a cognitive skill set for stimulus discrimination that is independent of a modality^46^. Yet, creating modal perceptual experience might require specific integration of both higher- and lower level sensory aspects, and thus potentially extensive periods of learning if the device is based on arbitrary coding schemes, if limits of neural plasticity on low-level sensory learning allow it at all. By contrast, our results suggest that mimicry of lower-level contingencies allowed our augmentation signal to integrate intuitively with existing perceptual processes and thereby transformed the nature of the learning problem from one of cross-modal plasticity to one of multi-sensory integration, here of vestibular integration.

Let us consider now how vestibular adaptation might have occurred. The vestibular literature distinguishes between the adaptation of the well-known subcortical pathways of the vestibular-ocular-reflex (VOR)^55^ and the calibration of perceived self-rotation. The perception of self-rotation has multiple, less-well known cortically-based underlying mechanisms that can be recalibrated independently of the VOR and have been shown to integrate signals from multiple sources^31^,^56^,^57^,^58^. Yet, while auditory cues support (illusory) self-motion perception^59^, to our knowledge there has been little evidence for an auditory contribution to the calibration of perceived vestibular self-rotation in the literature. By contrast, auditory localization is itself known to be a cortical process easily captured by other spatial signals^60^,^61^,^62^,^63^,^64^. In the ventroliquism effect for instance the perceived location of a sound source moves towards the location of a stable visual source such as a puppet’s mouth^60^. Similarly, auditory sources are displaced as an aftereffect following visual motion^62^, and the compression of visual space also leads to an accompanying compression of sound localization that moves the perceived location of the auditory source^61^. In particular, head movement signals, including vestibular signals, have a major influence on the localisation of directional sounds^65^,^66^,^67^.

Why then do our results show a change in the perception of vestibular space, as demonstrated by vestibular recalibration, if auditory-directional-cues are generally an unreliable source? We suggest that mimicry of auditory-directional contingencies allowed the augmentation signal to interface with existing vestibular-spatial processes, which then exploited the high spatial reliability of the magnetic-auditory sensorimotor contingency (providing an artificial allocentric reference) in a process of optimal multi-sensory integration, even though the auditory interface modality is not usually a major contributor to vestibular calibration. This is in line with the view that multimodal perception is driven by the reliability of the signals^68^,^69^ and of their convergence^40^,^41^,^42^, rather than by inherent physiological advantages of a modality^70^. While vision normally tends to dominate the perception of space, including vestibular calibration^35^,^58^, this role might be taken over by an artificial signal with equal (or higher) allocentric spatial reliability, provided that it is processed as a spatial signal by the perceptual system. Compatible with this claim, our behavioral results show that auditory information has led the vestibular system to recalibrate, rather than the other way around, and that it has done this with a similar magnitude as has been observed from re-calibration from optic flow^35^,^58^, which normally provides a calibrating spatial signal. Further, interviews with our participants revealed that for all except five of them (two in experiment 1, three in experiment 2), who report that they perceived it as an object rotating in space around them, the sound was immediately and reliably perceived as coming from a stable direction. These findings suggest that the sensorimotor processes maintaining spatial calibration have estimated the novel magnetic-auditory spatial reference as being world-stationary despite its bias and attributed its divergence with the vestibular information to internal-vestibular causes (errors in self-rotation estimation) rather than to external-auditory causes^71^, leading, potentially aided by priors for stationary external auditory sources^72^,^73^,^74^, to a recalibration of internal-vestibular rotation estimates, i.e. of vestibular spatial perception.

This opens the interesting prospect that a magnetic reference provided via auditory contingency-mimicry in a mobile device might lead the brain to intuitively utilize the stability of an artificial ‘magnetic reference’ for spatial calibration also in more natural behavior, with potential for improving navigation and for the compensation of spatial disabilities such as blindness or vestibular loss. Future studies might for example test if a magnetic-auditory allocentric reference of the head can diminish errors in perceived self-rotation during natural locomotion^75^,^76^,^77^. Further, a head-centered auditory reference might allow to compensate deficiencies in the allocentric calibration of vestibular position estimates in congenitally blind people^31^, contributing to the natural substitution of impaired vision by re-weighting of the remaining signals^78^, and similarly for vestibular loss^79^. It has been stressed that the development of effective prostheses should be informed by the science of sensory integration^80^,^81^ as well as the precise nature of the impairment^78^,^80^,^81^. Our contingency-mimetic method so far addresses the first point. It is based on sensory integration research suggesting that creating modal perception requires the integration of both low- and higher-level sensory features and applies the bio-mimetic principle at the level of sensory rather than neural contingencies, which in analogy we termed “contingency-mimetics”. Beyond the current auditory demonstration, another important future step is to apply contingency-mimetics also to other domains. In particular, tactile devices for sensory augmentation have been proposed signaling for instance whisker-like distance information to the head^82^,^83^ or hand^7^, or magnetic orientation^9^,^36^,^37^,^38^,^39^ or path information for wayfinding^84^,^85^,^86^,^87^,^88^ using waist-stimulating belts. However, as with traditional sensory substitution devices, these devices typically rely on comparatively arbitrary low-level tactile signal codings based on eccentric vibration motors, which do not provide the haptic profiles of natural tactile stimulation. In analogy to the role of HRTF in our auditory device, future tactile devices might thus do better by piggy-backing artificial sensors on ecological haptic contingencies^89^,^90^ such as slip motion^91^ or tactile flow^92^. We hypothesize that such contingency-mimetic tactile stimulation should then also lead to fast creation of modal perceptual experience and markedly reduce the required amount of learning and cognitive involvement^2^,^9^.

The idea of contingency mimetic sensory augmentation is inspired by the sensorimotor theory of conscious perception^29^,^93^, according to which conscious perceptual experience is constituted by knowledge and mastery of systematic changes in sensory input resulting from action, termed sensorimotor contingencies (SMCs). Mastery of SMCs may be established by relating sensory changes to motor actions ontogenetically during development and learning, or phylogenetically during evolution. Here we introduce the notions of higher-level and lower-level SMCs. Lower-level SMCs relate to changes in lower-order sensory signal properties which are putatively registered in early sensory neural processes; while higher-level SMCs relate to changes in higher-order sensory properties putatively registered in hierarchically higher-level neural processes. Following sensorimotor theory, mastery of *both* aspects of SMCs is constitutive of perceptual experience. Consequently, it is important how mastery of both aspects may be established. On the one hand, it is known that sensory development is driven by sensorimotor experience, even in primary sensory areas^94^,^95^, and that the brain is plastic throughout life^96^, suggesting that novel SMCs and perceptions can be learned during the life-span. On the other hand, sensory development, in particular in early areas, is affected by limits and critical periods of neural plasticity^97^,^98^ and genetic predetermination^99^. Therefore, if mastery of novel SMCs in arbitrary sensory codings requires a reformation of early sensory areas, this may pose strong physiological constraints on the SMCs that can be learned and the perceptual effects that can be created after adolescence, or even within a life span. Our results, in contrast, demonstrate that contingency-mimetic augmentation can provide novel sensory information as higher-level SMCs piggy-backed on a wide range of existing perceptually meaningful modal low-level SMCs. We argue that the novel higher-level SMCs are consequently subject to more higher-level cortical learning and adaptation with vastly higher plasticity and the potential to restructure and augment perception even in adulthood, dramatically reducing the challenge of learning novel SMCs for conscious perception, and thereby opening the prospect of truly perceptual sensory augmentation devices.

In summary, despite suggestions in the literature that sensory substitution and augmentation may never become truly perceptual^2^, and despite resulting questions about the practical use of sensory substitution^80^,^81^,^100^,^101^, our results demonstrate a truly modal perceptual impact of an artificial (magnetic North) signal provided via sensory augmentation, leading to a long-lasting impact on the metric of vestibular spatial experience. Our novel method is analogous to the bio-mimetic coding of artificial sensors within BMI approaches, which however have the disadvantage of being invasive. Our result shows that the mimetic principle, interfacing an artificial sensor by mimicking the natural characteristics of a neural signal, can be applied non-invasively at the sensory level as well. It is possible to feed an augmentation signal about magnetic North effectively into existing processes of multimodal space, thereby impacting perception, an approach that in analogy we term contingency-mimetic sensory augmentation.

## Methods

### Participants

#### Experiment 1

Twenty-three participants participated in the experiment in one of two groups. Thirteen participated in the group with smaller gain factor 0.5 (7 male, 2 author, mean age 33.7 years, std = deviation 11 years) and 14 in the group with larger gain factor 2 (6 male, 1 author, mean age 29 years, std = 5.8 years). Four participants participated in both conditions.

#### Experiment 2

Fifteen participants participated in the experiment in both gain conditions, measured on separate days (7 male, mean age 25 years, std = 8 years).

All participants gave written informed consent prior to participation. The study was approved by the Université Paris Descartes Review Board (CERES), and conducted in accordance with the Declaration of Helsinki. All participants, except the authors, were naïve to the purpose of the experiment.

### Setup

#### Sensory Augmentation Device

Our novel fully mobile augmentation device is based on a head-phone with integrated orientation sensors (compass, gyro, accelerometer) that are connected to an iPhone via BlueTooth LE (Fig. 1A,B). The iPhone app samples the current orientation with a frequency of 100 Hz and transforms the horizontal orientation of the head into a spatial sound using a 3D sound engine based on generic head-related transfer functions. The sound signals the direction of magnetic North within the head-based reference frame and moves in a compensatory manner to the movements of the head (i.e. in the opposite direction). The device has a low latency of 50ms and a spatial resolution of 1°.

#### Rotation Chair

Rotation experiments were performed on a motorized rotation chair at the Plateforme Sensorimotricité at University Paris Descartes. The rotation of the chair can be either set by the experimenter for passive rotation along trapezoidal velocity profiles, or participants can control the rotation via a rotation dial that directly controls the velocity of the chair during conditions of active rotation. During computer control, the motor (IRTSA Drives 2000/4000) is interfaced from Matlab via serial communication to set the parameters of a ramp generator using proportional–integral–derivative (PID) control. Prior to the experiment, we used a motion tracking system (Codamotion) to verify the angular precision of the rotation of the chair to be 2 degrees, which is more than accurate enough for our purpose here. The maximum rotational velocity is limited to 200°/s. Participants were under close visual surveillance during the entire experiment.

#### Angular Pointer

Participants indicated the perceived size of self-rotation using a custom made potentiometer-based angular pointer (Fig. 1C, similar to refs 34, 102. The pointer response was read out by an Arduino UNO board connected to the experiment computer via USB. The pointer’s sampling frequency is 40 Hz and the spatial resolution 0.8°. All responses where made starting at the pointer’s zero position, which was indicated by a small magnetic notch placed on the pointer handle so it could be sensed in the dark. To compensate for possible drift in the potentiometer’s zero voltage output within or across experiments, the pointer was calibrated by hand immediately prior to each experiment and response angles of each trial were calculated differentially as the voltage interval between start and end direction of the pointer.

### Procedure

#### Familiarization with Active Turning

Participants were blindfolded after sitting down on the rotation chair and given a 5-minute familiarization phase to accustom themselves with the velocity dial of the rotation chair before the experiment. In this, participants were initially asked to make full or half rotations, and then allowed to explore freely as they wished, while being encouraged to make use of the entire velocity range of the rotation chair.

#### Pointing Calibration

We trained the usage of the response pointer in the dark at the beginning of the experiment. Blind-folded participants were instructed over headphones to point to different locations across an imaginary clock centered at the middle of the pointing device (i.e. 30°, 60°, …, 330°). If their response was outside an interval of +−5°, they received auditory feedback and were asked to point again to the same location until they made a correct response. The accuracy of the initial pointing of a trial (i.e. without feedback) shows that participants can accurately indicate a desired direction with a mean SD of 12.8°.

#### Self-Rotation Experiment

Before and after the training phase, we tested participants’ vestibular perception of self-rotation using a vestibular adaptation experiment similar to the one developed by refs 34, 102. Blind-folded participants performed the rotation experiment in the absence of the auditory augmentation signal indicating North, only listening to a white-noise masking sound via the headphones. Vestibular stimulation was generated by passive whole-body rotation with displacements of 45°, 90°, 135° and 180° randomly to the left or the right. Rotations followed a triangular velocity profile with angular acceleration and deceleration of 11°/s^2^. Each angle was repeated 10 times, yielding a total of 80 trials. After each rotation, participants were asked to signal the size of the rotation by turning the angular pointer backwards to the start direction of the rotation. They were then asked to press a button to record the response, and to return the pointer back to its zero position for the next trial. A waiting period of 7 s was forced between two consecutive rotation onsets to rest the vestibular system between successive trials. In most trials the response execution itself already took longer than this required rest. Participants were not informed about the size of the possible rotations. Each new trial started at the end position of the previous trial. Instructions were given verbally during the experiment via the headphones using text-to-speech synthesis.

#### Post Interviews

After the entire experiment, participants were asked to note anything unusual or unpleasant during the experiment and how they felt the movements, whether they heard the sound as moving around themselves or as always coming from a fixed direction, and how they reached the desired turn size.

### Analysis

#### Linear Mixed Effect Models

In both experiments, we performed a separate linear mixed effects analysis for each gain group on the relation between self-rotation estimates and time using MATLAB and the Statistics Toolbox (Release 2016a, The MathWorks, Inc., Natick, Massachusetts, United States). We included stimulus angle A, time T (baseline, post training), and the interaction between stimulus angle and time (A:T) both as fixed effects (β) and as random effects grouped by participants (*u*) to characterize changes in intercept and slope at the individual as well as the group level:


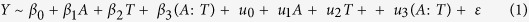


In experiment 2, we additionally tested long-lasting recalibration effects by fitting separate models for each gain on the relation between response angles of the pre-training measurements and the testing session S (day 1, day 2) to test if training on day 1 affected the baseline on day 2:


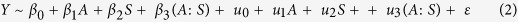


All models were fitted using the default maximum-likelihood procedure of the *fitlme* method. Fixed effects were tested using Wald statistics. Model comparison was performed using a likelihood ratio test via the *compare* method. Turning directions were collapsed before analysis.

### Data Availability

The data that support the findings of this study are available from the corresponding author upon reasonable request.

## Additional Information

**How to cite this article**: Schumann, F. and O’Regan, J. K. Sensory augmentation: integration of an auditory compass signal into human perception of space. *Sci. Rep.* **7**, 42197; doi: 10.1038/srep42197 (2017).

**Publisher's note:** Springer Nature remains neutral with regard to jurisdictional claims in published maps and institutional affiliations.

## Figures and Tables

**Table 1:** Fixed Effect Parameters for Experiment 1, using Equation 1.

Parameter	Estimate		SE		t value		p value	
	gain 0.5	gain 2	gain 0.5	gain 2	gain 0.5	gain 2	gain 0.5	gain 2
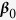 **(intercept)**	20.347	11.873	5.212	5.33	3.903	2.22	<0.0001	0.025
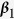 **(Angle)**	0.794	1.108	0.107	0.090	7.391	12.276	<0.0001	<0.0001
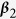 **(Time - Post Training)**	7.487	27.537	4.914	6.401	1.523	4.235	0.127	<0.0001
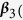 **Interaction)**	−0.211	0.070	0.056	0.060	−3.741	1.177	0.0002	0.239

**Table 2:** Fixed Effect Parameters for Experiment 2, using Equation 1.

Parameter	Estimate		Std. Error		t value		p value	
	gain 0.5	gain 2	gain 0.5	gain 2	gain 0.5	gain 2	gain 0.5	gain 2
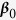 **(intercept)**	15.975	13.517	6.089	3.373	2.623	4.006	0.009	<0.0001
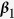 **(Angle)**	1.148	0.8713	0.076	0.074	14.984	11.69	<0.0001	<0.0001
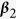 **(Time - Post Training)**	4.599	10.669	5.069	4.489	0.907	2.376	0.364	0.017
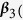 **Interaction)**	−0.416	0.278	0.061	0.056	−6.805	4.994	<0.0001	<0.0001

**Table 3:** Fixed Effect Parameters Testing Long-Lasting Recalibration in Experiment 2, using Equation 2.

Parameter	Estimate		Std. Error		t value		p value	
	gain 0.5	gain 2	gain 0.5	gain 2	gain 0.5	gain 2	gain 0.5	gain 2
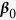 (intercept)	13.59	11.539	7.034	5.742	1.932	2.009	0.536	0.044
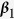 (Angle)	1.011	1.0463	0.088	0.080	11.429	13.035	<0.0001	<0.0001
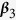 (Session – Day 2)	2.3594	6.4389	6.417	8.211	0.367	0.784	0.713	0.433
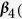 Interaction)	−0.3419	0.22305	0.082	0.070	−4.136	3.15	<0.0001	0.002
