# Corrigendum: Sensory augmentation: integration of an auditory compass signal into human perception of space

**DOI:** 10.1038/srep44965

**Published:** 2017-03-23

**Authors:** Frank Schumann, J. Kevin O’Regan

Scientific Reports 7: Article number: 4219710.1038/srep42197; published online: 02 14 2017; updated: 03 23 2017

This Article contains an error in the Acknowledgements section:

“Matthieu Raoelsion”

should read:

“Matthieu Raoelison”

