# Erratum: Sensory augmentation: integration of an auditory compass signal into human perception of space

**DOI:** 10.1038/srep46810

**Published:** 2018-01-17

**Authors:** Frank Schumann, J. Kevin O’Regan

Scientific Reports 7: Article number: 42197; 10.1038/srep42197 published online: 02 14 2017; updated: 01 17 2018.

Due to a typesetting error, the original version of this Article contained errors in the Reference list and in-text citations. References 39 to 102 were incorrectly listed as 40 to 103. In addition, reference 103 was incorrectly listed as 39. 

In the Discussion section,

“Contrary to tactile waist-stimulation approaches^9,36,37,38,39^, additionally, the magnetic information is presented close to real time and shares the reference frame of the eyes, ears and vestibular equilibration, favoring temporal and spatial integration^39,40,41,42^.”

now reads:

“Contrary to tactile waist-stimulation approaches^9,36,37,38,103^, additionally, the magnetic information is presented close to real time and shares the reference frame of the eyes, ears and vestibular equilibration, favoring temporal and spatial integration^39,40,41,42^.”

“The perception of self-rotation has multiple, less-well known cortically-based underlying mechanisms that can be recalibrated independently of the VOR and have been shown to integrate signals from multiple sources^31,56–58^.”

now reads:

“The perception of self-rotation has multiple, less-well known cortically-based underlying mechanisms that can be recalibrated independently of the VOR and have been shown to integrate signals from multiple sources^32,56–58^.”

“In particular, tactile devices for sensory augmentation have been proposed signaling for instance whisker-like distance information to the head^82,83^ or hand^7^, or magnetic orientation^9,36,37,38,39^ or path information for wayfinding^84,85,86,87,88^ using waist-stimulating belts.”

now reads:

“In particular, tactile devices for sensory augmentation have been proposed signaling for instance whisker-like distance information to the head^82,83^ or hand^7^, or magnetic orientation^9,36,37,38,103^ or path information for wayfinding^84,85,86,87,88^ using waist-stimulating belts.”

“The idea of contingency mimetic sensory augmentation is inspired by the sensorimotor theory of conscious perception^29,93^, according to which conscious perceptual experience is constituted by knowledge and mastery of systematic changes in sensory input resulting from action, termed sensorimotor contingencies (SMCs).”

now reads:

“The idea of contingency mimetic sensory augmentation is inspired by the sensorimotor theory of conscious perception^28,93^, according to which conscious perceptual experience is constituted by knowledge and mastery of systematic changes in sensory input resulting from action, termed sensorimotor contingencies (SMCs).”

Furthermore in the Methods section under the subheading ‘Angular Pointer’,

“Participants indicated the perceived size of self-rotation using a custom made potentiometer-based angular pointer (Fig. 1C, similar to refs 34, 102).”

now reads:

“Participants indicated the perceived size of self-rotation using a custom made potentiometer-based angular pointer (Fig. 1C, similar to refs 35, 102).”

In the Methods section under the subheading ‘Self-Rotation Experiment’:

“Before and after the training phase, we tested participants’ vestibular perception of self-rotation using a vestibular adaptation experiment similar to the one developed by refs 34, 102.”

now reads:

“Before and after the training phase, we tested participants’ vestibular perception of self-rotation using a vestibular adaptation experiment similar to the one developed by refs 35, 102.”

In addition, this Article contained errors in the following equations:

In Equation 1:
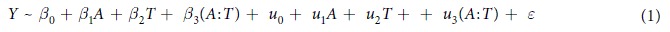


now reads:


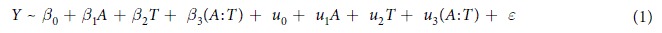


In Equation 2,
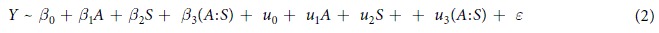


now reads:


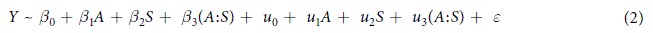


These errors have now been corrected in the PDF and HTML versions of the Article.

